# Migratory Birds Reinforce Local Circulation of Avian Influenza Viruses

**DOI:** 10.1371/journal.pone.0112366

**Published:** 2014-11-12

**Authors:** Josanne H. Verhagen, Jacintha G. B. van Dijk, Oanh Vuong, Theo Bestebroer, Pascal Lexmond, Marcel Klaassen, Ron A. M. Fouchier

**Affiliations:** 1 Department of Viroscience, Erasmus MC, Rotterdam, The Netherlands; 2 Department of Animal Ecology, Netherlands Institute of Ecology (NIOO-KNAW), Wageningen, The Netherlands; 3 Centre for Integrative Ecology, School of Life and Environmental Sciences, Deakin University, Geelong, Australia; Justus-Liebeig University Giessen, Germany

## Abstract

Migratory and resident hosts have been hypothesized to fulfil distinct roles in infectious disease dynamics. However, the contribution of resident and migratory hosts to wildlife infectious disease epidemiology, including that of low pathogenic avian influenza virus (LPAIV) in wild birds, has largely remained unstudied. During an autumn H3 LPAIV epizootic in free-living mallards (*Anas platyrhynchos*) — a partially migratory species — we identified resident and migratory host populations using stable hydrogen isotope analysis of flight feathers. We investigated the role of migratory and resident hosts separately in the introduction and maintenance of H3 LPAIV during the epizootic. To test this we analysed (i) H3 virus kinship, (ii) temporal patterns in H3 virus prevalence and shedding and (iii) H3-specific antibody prevalence in relation to host migratory strategy. We demonstrate that the H3 LPAIV strain causing the epizootic most likely originated from a single introduction, followed by local clonal expansion. The H3 LPAIV strain was genetically unrelated to H3 LPAIV detected both before and after the epizootic at the study site. During the LPAIV epizootic, migratory mallards were more often infected with H3 LPAIV than residents. Low titres of H3-specific antibodies were detected in only a few residents and migrants. Our results suggest that in this LPAIV epizootic, a single H3 virus was present in resident mallards prior to arrival of migratory mallards followed by a period of virus amplification, importantly associated with the influx of migratory mallards. Thus migrants are suggested to act as local amplifiers rather than the often suggested role as vectors importing novel strains from afar. Our study exemplifies that a multifaceted interdisciplinary approach offers promising opportunities to elucidate the role of migratory and resident hosts in infectious disease dynamics in wildlife.

## Introduction

Migratory and resident (i.e. sedentary) hosts are thought to fulfil different, non-mutually exclusive, roles in infectious disease dynamics in wild animal populations, although empirical evidence is largely lacking. For one, migratory hosts may transport pathogens to new areas, resulting in the exposure and potential infection of new host species, thereby contributing to the global spread of infectious diseases [1]. Resident hosts, immunologically naïve to these novel pathogens, may subsequently act as local amplifiers. For instance, the global spread of West Nile Virus (WNV) is considered to be greatly facilitated by migratory birds introducing the virus to other wildlife and humans in many parts of the world [2]. Similarly, the introduction of Ebola virus into humans in the Democratic Republic of Congo, Africa, in 2007 coincided with massive annual fruit bat migration [3].

Additionally, migratory hosts may amplify pathogens upon arrival at a staging site, either because they are immunologically naïve to locally circulating pathogens [4] and/or as a consequence of reduced immunocompetence due to the trade-off between investment in immune defences and long-distance flight [1]. Correspondingly, pathogen prevalence or the risk of disease outbreaks may locally be reduced when migratory hosts depart [1]. Consistent with the role for migrants, residents in this scenario are suggested to act as reservoirs, permanently maintaining pathogens within their population and transmitting them to other hosts, including migrants [5], [6]. Given these potentially distinct roles for migratory and resident hosts in the spatial and temporal spread of infectious diseases, it is important to differentiate between migratory and resident hosts when aiming to improve our understanding of the ecology, epidemiology, and persistence of diseases in wild animal populations.

Wild bird populations are considered the reservoir hosts of low pathogenic avian influenza A viruses (LPAIV). Predominantly birds from wetlands and aquatic environments (orders *Anseriformes* and *Charadriiformes*) are infected with LPAIV [7], causing transient and mainly intestinal infections [8], [9], with no or limited signs of disease [10]. LPAIV can be classified in subtypes based on antigenic and genetic variation of the viral surface glycoproteins hemagglutinin (HA) and neuraminidase (NA). All subtypes that have been recognized to date, notably HA subtypes 1 through 16 (H1-H16) and NA subtypes 1 through 9 (N1-N9), have been found in wild birds [11]. Recently, novel influenza viruses were identified in fruit bats that are distantly related to LPAIV (H17N10, H18N11), indicating that bats, alongside wild birds, harbour influenza viruses and might play a distinct role in the dynamics of this infectious disease [12], [13].

Despite a large number of studies on the ecology and epidemiology of LPAIV in wild birds, only few studies have focussed on the role of resident and migratory hosts in the dynamics of this infectious disease. Resident bird species likely facilitate LPAIV transmission, while migratory bird species harbour high LPAIV subtype diversity after arrival at the wintering grounds [14], [15]. In most of these studies resident and migratory hosts belonged to different bird species, with presumably different LPAIV susceptibility. However, many bird species are composed of a mixture of resident and migratory individuals, so called partial migrants [16]. Individuals that belong to the same species but use distinct migratory strategies, may differ in morphology and behaviour (e.g. body size, dominance; [17]), immune status and pathogen exposure. As a consequence, resident and migratory individuals of a single species may respond differentially to LPAIV infection and hence their contribution to local, and consequently global, LPAIV infection dynamics may differ. Hill et al. investigated the role of migratory and resident hosts of a single bird species in LPAIV infection dynamics. In their study, no differences were detected in LPAIV prevalence between migratory and resident host populations [18]. However, migrants likely introduced LPAIV subtypes from their breeding areas to the wintering grounds and residents likely acted as LPAIV reservoirs facilitating year-round circulation of limited subtypes [18]. A similar study in the same species conducted at a local scale instead of a macro-ecological scale, showed that susceptible migratory hosts were more frequently infected with LPAIV than residents, which had probably driven the epizootic in autumn [19]. LPAIV epizootics in wild birds are likely to take place at local spatial and temporal scales, since LPAIV infections are generally short (i.e. up to a week; [20]), and most virus particles are shed within the first few days after infection [21]. Yet, the precise role of migratory and resident hosts during local LPAIV epizootics in terms of virus introduction and reinforcement, including host immunity, has remained largely unstudied.

We build on the study of van Dijk et al. [19] to investigate the role of migratory and resident hosts of a single bird species during a local LPAIV epizootic. Throughout an H3 LPAIV epizootic at the wintering grounds in autumn 2010, we sampled a partly migratory bird species, the mallard (*Anas platyrhynchos*), and connected host migratory strategy with (i) H3 virus kinship, (ii) H3 virus prevalence and shedding, and (iii) H3-specific antibody prevalence. H3 LPAIV is a dominant subtype in wild ducks in the northern hemisphere [22], [23]. This study provides a detailed description of a monophyletic H3 LPAIV epizootic importantly associated with the influx of migratory mallards.

## Materials and Methods

### Ethics statement

Capturing free-living mallards was approved by the Dutch Ministry of Economic Affairs based on the Flora and Fauna Act (permit number FF/75A/2009/067 and FF/75A/2010/011). Handling and sampling of free-living mallards was approved by the Animal Experiment Committee of the Erasmus MC (permit number 122-09-20 and 122-10-20) and the Royal Netherlands Academy of Arts and Sciences (KNAW) (permit number CL10.02). Free-living mallards were released into the wild after sampling. All efforts were made to minimize animal suffering throughout the studies.

### Study species and site

Mallards are considered a key LPAIV host species, together with other dabbling duck species of the *Anas* genus, harbouring almost all LPAIV subtype combinations found in birds to date [11]. Mallards are partially migratory, meaning that the population exists of both migratory and resident birds. Along the East Atlantic Flyway, mallards breeding in Scandinavia, the Baltic, and northwest Russia migrate to winter at more southern latitudes in autumn, congregating with the resident populations that breed in Western Europe, including the Netherlands [24].

During the 2010 LPAIV epizootic described here, free-living mallards were caught in swim-in traps of a duck decoy [25]. The duck decoy was located near Oud Alblas (51°52′38″N, 4°43′26″E), situated in the province of Zuid-Holland in the Netherlands. This sampling site is part of the ongoing national wild bird avian influenza virus (AIV) surveillance program (dd 2014-09-20), executed by the department of Viroscience of Erasmus MC, where mallards, free-living and hunted in the near surrounding, were sampled for LPAIV from 2005 onwards.

### Sampling

During the LPAIV epizootic (i.e. from August until December 2010) studied here, the duck decoy was visited, on average, seven times per month capturing approximately 11 birds per visit. Each captured mallard was marked using a metal ring with an unique code, aged (juvenile: <1 year, adult:>1 year) and sexed based on plumage characteristics [26]. For virus detection, cloacal and oropharyngeal samples were collected using sterile cotton swabs as LPAIV may replicate in both the intestinal and respiratory tract of wild birds [27]. Swabs were stored individually in virus transport medium (Hank's balanced salt solution with supplements; [28]) at 4°C, and transported to the laboratory for analysis within seven days of collection. For detection of antibodies to AIV, blood samples (<1 ml, 2% of the circulating blood volume) were collected from the brachial vein, which were allowed to clot for approximately 6 h before centrifugation to separate serum from red blood cells [29]. Serum samples were stored at −20°C until analysis. To determine a bird's migratory strategy using stable hydrogen isotope analysis, the tip (1–2 cm) of the first primary feather of the right wing was collected and stored in a sealed bag at room temperature. Of recaptured birds, both swabs and a blood sample were collected.

### Migratory strategy

In the study of van Dijk et al. [19], the origin (and hence, migratory strategy) of mallards sampled during the 2010 LPAIV epizootic was determined using stable hydrogen isotope analysis in feathers. Stable isotope signatures in feathers reflect those of local food webs [30]. During the period of growth (i.e. moult), local precipitation is incorporated into these feathers [31], causing the stable hydrogen isotope (δ^2^H) ratio in feathers to be correlated with δ^2^H of local precipitation [32]. Across Europe, a gradient of δ^2^H in feathers is found in mallards [33]. Based on feather δ^2^H and additional criteria, van Dijk et al. [19] classified mallards as resident, local migrant (i.e. short distance) and distant migrant (i.e. long distance). A resident bird had grown its feathers near the duck decoy (was captured during moult) and was recaptured multiple times either before or during the LPAIV epizootic. A local and distant migratory bird was seen and sampled once, i.e. only during the LPAIV epizootic and was not captured within one year before this epizootic. Based on feather δ^2^H values of local (−103.5 to −72.6‰) and distant migrants (−164.5 to −103.7‰) and using a European feather δ^2^H isoscape of mallards [33], local migrants originated roughly from central Europe and distant migrants roughly from north-eastern Europe. We used similar criteria to assess the migratory strategy of mallards caught during the H3 LPAIV epizootic. For 149 individual birds in this study we were unable to assign them to either the resident or migratory population and these were excluded from analyses, except the genetic analysis.

For full details on the stable hydrogen isotope analysis, see van Dijk et al. [33]. In short, feathers were cleaned and air-dried overnight. Feather samples were placed into silver capsules, stored in 96 well trays and shipped to the Colorado Plateau Stable Isotope Laboratory (Northern Arizona University, Flagstaff, USA). Stable hydrogen isotope analyses were performed on a Delta Plus XL isotope ratio mass spectrometer equipped with a 1400 C TC/EA pyrolysis furnace. Feather δ^2^H values are reported in units per mil (‰) relative to the Vienna Standard Mean Ocean Water-Standard Light Antarctic Precipitation (VSMOW-SLAP) standard scale.

### Virus detection, isolation and characterization

As part of the national wild bird AIV surveillance program — including the 2010 LPAIV epizootic — LPAIV infection of free-living and hunted mallards was assessed using cloacal and oropharyngeal swab samples. RNA from these samples was isolated using the MagnaPure LC system with a MagnaPure LC total nucleic acid isolation kit (Roche Diagnostics, Almere, the Netherlands) and analysed using a real-time reverse transcriptase-PCR (RT-PCR) assay targeting the matrix gene. Matrix RT-PCR positive samples were used for the detection of H5 and H7 influenza A viruses using HA specific RT-PCR tests [28], [34]. All matrix positive samples were used for virus isolation in embryonated chicken eggs and characterized as described previously [28].

Matrix RT-PCR positive samples collected during the 2010 LPAIV epizootic for which virus culture was not successful, were screened for the presence of H3 influenza A viruses using a H3 specific RT-PCR test (n = 126). Additionally, matrix RT-PCR positive samples collected half year prior to the LPAIV epizootic (November 2009-July 2010) were screened for the presence of H3 influenza A viruses to determine whether H3 LPAIV was detected in mallards prior to the epizootic (n = 20). Amplification and detection were performed on an ABI 7500 machine with the taqman Fast Virus 1 Step Master mix reagents (Applied Biosystems, Nieuwerkerk aan den IJssel, the Netherlands) and 5 µl of eluate in an end volume of 30 µl using 10 pmol Oligonucleotides RF3226 (5′-GAACAACCGGTTCCAGATCAA -3′) and 40 pmol RF3227 (5′- TGGCAGGCCCACATAATGA-3′) and 10 pmol of the double-dye labelled probe RF3228 (5′-FAM-TCCTRTGGATTTCCTTTGCCATATCATGC-BHQ-3′). Primers and probe were designed with the software package Primer Express version 3.01 (Applied Biosystems, Nieuwerkerk aan den IJssel, the Netherlands), based on avian H3 nucleotide sequences obtained from Genbank (www.ncbi.nlm.nih.gov).

The degree of virus shedding from the cloaca and the oropharynx during the LPAIV epizootic was based on the cycle threshold (C_T_) value, i.e. first real-time matrix RT-PCR amplification cycle in which matrix gene amplification was detected. The C_T_-value is inversely proportional to the amount of viral RNA in a sample.

### Sequence analysis and phylogeny

To investigate H3 LPAIV diversity in time and space among resident and migratory mallards during the LPAIV epizootic, we performed a genetic analysis focussed on the HA segment, one of the two most variable gene segments of LPAIV. Nucleotide sequences of the HA gene segment were obtained from virus isolates that were previously characterized by hemagglutination inhibition (HI) assay as H3 LPAIV. The RT-PCR and sequencing of the HA segment was performed using HA specific primers (5′- GGATCTGCTGCTTGTCCTGT-3′ and 5′- GRATAAGCATCTATTGGAC-3′), as described previously [35].

A total of 86 HA gene segments of 1576 nt in length were included in the genetic analysis. The genetic analysis comprised H3 nucleotide sequences obtained from (i) residents and migratory mallards during the 2010 LPAIV epizootic (n = 23), (ii) additional H3 LPAIV isolates from the national wild bird surveillance program of Erasmus MC (n = 35), and (iii) a BLAST analysis using public databases available as of 29 November 2013 (www.ncbi.nlm.nih.gov, http://www.gisaid.com), from which only European virus sequences with a known isolation date were retrieved (n = 28). Duplicate and incomplete sequences were removed. Nucleotide sequences were aligned using the software MAFFT version 7 (http://mafft.cbrc.jp/alignment/software/).

H3 nucleotide sequences were labelled based on sampling site, year of virus isolation, and host migratory strategy (i.e. resident, local migrant, distant migrant). During the 2010 LPAIV epizootic, H3 nucleotide sequences were obtained from 23 viruses, isolated from residents (n = 3), from local migrants (n = 13), from distant migrants (n = 2), and from birds of which the migratory strategy could not be assessed (n = 5). This was supplemented with 12 H3 nucleotide sequences obtained from viruses isolated from mallards sampled in the duck decoy in different years, notably in 2008 (n = 11) and 2011 (n = 1). There were 31 H3 nucleotide sequences from virus samples collected at other sampling locations in the Netherlands and elsewhere in Europe between 1999 and 2011. Of these virus samples, 18 originated from locations within the province of Zuid-Holland (5–30 km from the duck decoy), i.e. from Berkenwoude (n = 13) (51°57′00″N, 4°41′36″E), Lekkerkerk (n = 2) (51°53′41″N, 439′24″E), Oudeland van Strijen (n = 2) (51°46′56″N, 4°30′56″E) and Vlist (n = 1) (51°59′13″N, 4°45′56″E). Eleven viruses were isolated from birds in coastal regions in the Netherlands (i.e. 115–200 km from the duck decoy), i.e. Schiermonnikoog (n = 1) (53°28′41″N, 6°9′24″E), Vlieland (n = 1) (53°16′42″N, 5°1′22″E), Westerland (n = 8) (52°53′39″N, 4°56′32″E) and Wieringen (n = 1) (52°54′0″N, 4°58′11″E). Outside the Netherlands, two H3 sequences were from viruses isolated in Hungary in 2009. The remaining 20 H3 nucleotide sequences originated from multiple locations throughout Europe (i.e. Belgium, Czech Republic, Germany, Iceland, Italy and Switzerland) and Russia.

A Maximum Likelihood (ML) phylogenetic tree was generated using the PhyML package version 3.1 using the GTR+I+G model of nucleotide substitution, performing a full heuristic search and subtree pruning and regrafting (SPR) searches. The best-fit model of nucleotide substitution was determined with jModelTest [36]. Tree was visualized using the Figtree program, version 1.4.0 (http://tree.bio.ed.ac.uk/software/figtree). Overall rates of evolutionary change (i.e. number of nucleotide substitutions per site per year) and time of circulation to the most recent common ancestor (TMRCA) in years was estimated using the BEAST program version 1.8.0 (http://beast.bio.ed.ac.uk/). To accommodate variation in the molecular evolutionary rate among lineages, the uncorrelated log-normal relaxed molecular clock was used. Isolation dates were used to calibrate the molecular clock. Three independent Bayesian Markov Chain Monte Carlo (MCMC) analyses were performed for 50 million states, with sampling every 2,000 states. Convergence and effective sample sizes of the estimate were checked with Tracer v1.6 (http://tree.bio.ed.ac.uk/software/tracer/). Uncertainty in parameter estimates was reported as the 95% highest posterior density (HPD) [37]. Nucleotide sequences are online available under the accession numbers as listed in Table S1 and S2

### Serology

To assess whether mallards had H3-specific antibodies during the 2010 LPAIV epizootic, all sera were first tested for the presence of AIV antibodies specific for the nucleoprotein (NP) using a multispecies blocking enzyme-linked immunosorbent assay (bELISA MultiS-Screen Avian Influenza Virus Antibody Test Kit; IDEXX Laboratories, Hoofddorp, the Netherlands), following manufacturer's instructions. Each plate contained two positive and two negative controls. Samples were tested in duplicate. An infinite M200 plate reader (Tecan Group Ltd, Männedorf, Switzerland) was used to measure the absorbance (i.e. OD-value) at 620 nm. Samples were considered positive for the presence of NP antibodies when signal-to-noise ratios (i.e. mean OD-value of the sample divided by the mean OD-value of the negative control) were <0.5. NP antibody positive serum samples were subsequently tested for the presence of H3-specific antibodies using the HI assay according to standard procedures [38]. Briefly, sera were pre-treated overnight at 37°C with receptor destroying enzyme (Vibrio cholerae neuraminidase) and incubated at 56°C for 1 h. Two-fold serial dilutions of the antisera, starting at a 1∶10 dilution, were mixed with 4 hemagglutinating units of A/Mallard/Netherlands/10/2010 (H3N8) in 25 µl and were incubated at 37°C for 30 min. Subsequently, 25 µl 1% turkey erythrocytes was added and the mixture was incubated at 4°C for 1 h. Hemagglutination inhibition patterns were read and the HI titre was expressed as the reciprocal value of the highest dilution of the serum that completely inhibited agglutination of turkey erythrocytes.

### Statistics

Birds were considered LPAIV positive when either cloacal or oropharyngeal swabs were positive. To exclude samples of birds that had been sampled twice within the same infectious period during the 2010 LPAIV epizootic, we used an interval of at least 30 days between the day that a bird tested LPAIV positive and the next sampling day. Mallards may shed virus up to 18 days [21].

During the LPAIV epizootic, 709 cloacal and oropharyngeal swabs were collected from 472 mallards of which 129 individuals were recaptured. Of these swabs, 84 tested positive for H3 LPAIV, 35 tested LPAIV positive but H3 negative (i.e. matrix-positive H3-negative), and 583 swabs tested LPAIV negative. Of 7 matrix-positive swabs we were unable to determine H3-positivity. To test H3 virus prevalence and shedding, we included H3-positive and H3-negative swabs (i.e. matrix-negative and matrix-positive). Swabs from birds of which the migratory strategy could not be assessed (n = 269) or with undefined age and sex (n = 13) were excluded. The exclusion of birds of which the migratory strategy could not be assessed did not affect the temporal pattern of H3 LPAIV prevalence. In total we included 420 cloacal and oropharyngeal swabs from 305 individual birds, of which 55 birds were sampled more than once (Table S3).

During the LPAIV epizootic, 428 serum samples were collected from 364 mallards of which 52 individuals were recaptured. Of these serum samples, 9 tested positive for H3-specific antibodies, 98 tested positive for AIV antibodies but negative for H3-specific antibodies (i.e. NP-positive H3-negative), and 321 sera tested negative for AIV antibodies. To investigate H3-specific antibody prevalence, we included H3-specific antibody positive and H3-specific antibody negative sera (i.e. NP-negative and NP-positive). Sera from birds of which the migratory strategy could not be assessed (n = 96) or with undefined age and sex (n = 5) were excluded. Thus in total we included 320 sera samples from 281 individual birds, of which 30 birds were sampled more than once (Table S3).

A generalized linear mixed model (GLMM) was used in the analysis of H3 virus prevalence, with migratory strategy (i.e. resident, local migrant, distant migrant), age, sex and month as fixed factors, all two-way interactions with migratory strategy, and individual bird as random factor. The interactions between migratory strategy and age, migratory strategy and sex, and migratory strategy and month were tested to assess whether H3 virus prevalence differed per age class, sex and month for the three categories of migratory strategy. The fixed factors age and sex were merely included in the models to conduct the interactions. A general linear model (GLM) was used to test for differences in prevalence of H3-specific antibodies, with migratory strategy and month as fixed factors. Linear models (LMs) were used to determine differences in the degree of virus shedding of H3 LPAIV-particles based on viral RNA from the cloaca and the oropharynx (i.e. C_T_-value) with migratory strategy and month as fixed factors. A Tukey's post hoc test was performed to detect differences in H3 LPAIV prevalence between the three categories of migratory strategy and months. All analyses were conducted using R 2.14.1 [39]. Package lme4 was used to fit the GLMM [40] and multcomp to perform a Tukey's post hoc test [41].

## Results

### Virus prevalence

**Figure 1:**
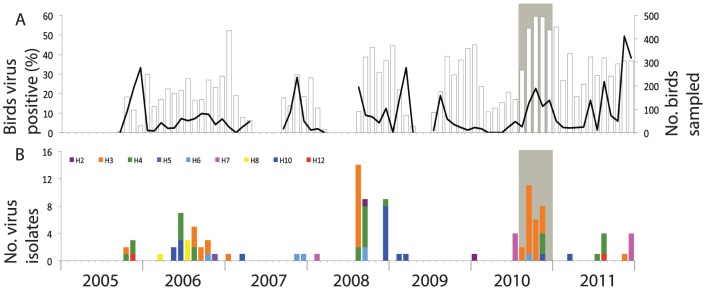
Prevalence and subtype diversity of low pathogenic avian influenza viruses (LPAIV) in mallards sampled at Oud Alblas, the Netherlands, 2005–2011. The grey-shaded area indicates the H3 LPAIV epizootic from August until December 2010. (A) Number of free-living and hunted birds sampled (bars, right Y-axis) and percentage of birds tested virus positive based on M RT-PCR (line, left Y-axis). (B) Number of virus isolates per HA subtype: H2 (purple), H3 (orange), H4 (green), H5 (light purple), H6 (light blue), H7 (pink), H8 (yellow), H10 (dark blue) and H12 (red).

Each year, from 2005 until 2011, LPAIV prevalence in mallards peaked between the end of summer (August) and the beginning of winter (December), with some exceptions in March 2009 and June 2011 (Figure 1A). Detection of the various HA subtypes varied per year, with most virus isolates found in autumn, notably H2 to H8, H10, and H12. H3 LPAIV was isolated from mallards every year, except in 2007 and 2009, and was the dominant HA subtype in 2006, 2008 and 2010 (Figure 1B).

**Figure 2:**
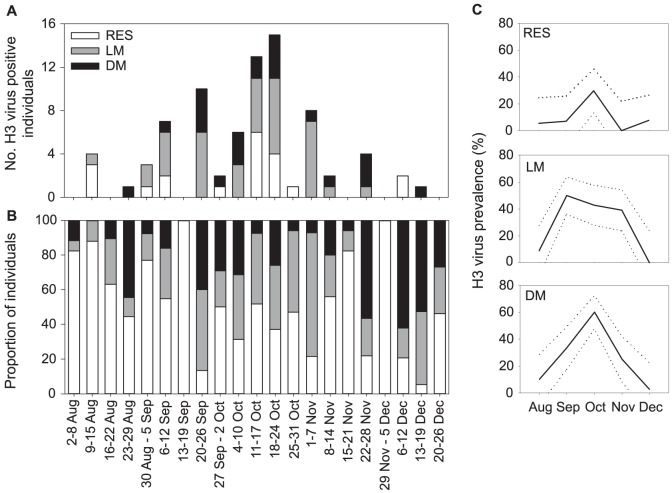
Prevalence of H3 low pathogenic avian influenza viruses (LPAIV) in residents, local and distant migratory mallards during the H3 LPAIV epizootic in 2010. For residents (RES), local migrants (LM) and distant migrants (DM) the (A) number of H3 virus positive individuals per week, (B) proportion of individuals sampled per week, and (C) H3 virus prevalence (±95% CI) per month are depicted.

During the 2010 LPAIV epizootic, mallards were infected with H3 LPAIV (84 of 709, 12%) and with other LPAIV subtypes, namely H4, H6 and H10 (35 of 709, 5%; Figure 1B). The H3 LPAIV epizootic started on the 12^th^ of August 2010 (Figure 2A) and H3 virus prevalence differed between months (Table 1). H3 virus prevalence increased in September, peaked in October, and decreased in November and December (Figure 2A and 2C). Shortly before the 2010 LPAIV epizootic, a single mallard of unknown origin was infected with H3 LPAIV on the 10^th^ of February 2010, followed by a period of five months where no H3 infections were detected among 536 mallards sampled.

**Table 1:** Linear model test results of the analysis of H3 low pathogenic avian influenza virus (LPAIV) prevalence during the LPAIV epizootic in 2010.

	H3 virus prevalence	
Variable	X^2^	p-value
Age	0.144	0.705
Sex	0.659	0.417
Month	44.928	**<0.001**
Migratory strategy	23.681	**<0.001**
Migratory strategy * Age	0.777	0.678
Migratory strategy * Sex	0.558	0.757
Migratory strategy * Month	21.510	**0.006**

Besides migratory strategy, age, sex, month and two-way interactions were included. Significant values (p<0.05) are shown in bold.

Local and distant migrants were more often infected with H3 LPAIV (37 of 113, 33% and 22 of 98, 22% respectively) than residents (20 of 209, 10%; Figure 2C, Table 1). The peak month of the H3 LPAIV epizootic differed between the three mallard populations (Table 1): in local migrants H3 LPAIV infection peaked in September, whereas in residents and distant migrants infection peaked in October (Figure 2C). At the start of the H3 LPAIV epizootic (12^th^ of August), three residents and one local migrant were infected with H3 LPAIV, with their populations constituting respectively 88% and 12% of the sampled mallard population. Two weeks later (26^th^ of August), the first distant migrant infected with H3 LPAIV was detected (44% of the sampled mallard population). In September and October, most mallards infected with H3 LPAIV were local migrants (respectively 12 of 22 and 15 of 35 total H3 LPAIV positives), while local migrants comprised respectively 24% and 40% of the sampled mallard population. In October, 11 residents and nine distant migrants were infected with H3 LPAIV, the latter constituting only 17% of the sampled mallard population. In November, only nine local and five distant migrants were infected with H3 LPAIV (comprising respectively 29% and 25% of the sampled mallard population). The last month of the H3 LPAIV epizootic, only one distant migrant and two residents were infected with H3 LPAIV, although distant migrants and residents constituted respectively 43% and 32% of the sampled mallard population.

### Virus shedding

H3 virus shedding from the cloaca and oropharynx did not differ between the three mallard populations (F_2,10_ = 1.051, p = 0.385 and F_2,63_ = 0.025, p = 0.976, respectively). Nor were there any differences in the monthly amount of H3 virus shed from the cloaca and oropharynx during the H3 LPAIV epizootic (F_3,10_ = 1.945, p = 0.186 and F_4,63_ = 1.124, p = 0.353, respectively).

### Antibody prevalence

**Figure 3:**
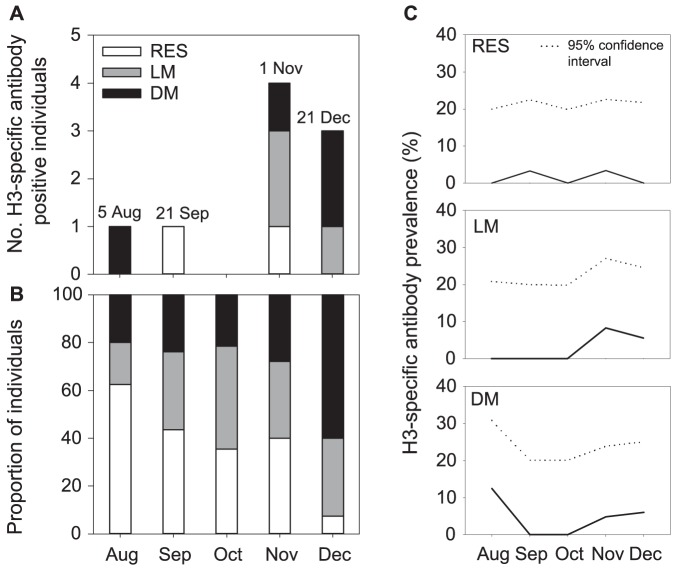
Prevalence of avian influenza H3-specific antibodies in residents, local and distant migratory mallards during the H3 low pathogenic avian influenza virus (LPAIV) epizootic in 2010. For residents (RES), local migrants (LM) and distant migrants (DM) the (A) number of H3-specific antibody positive individuals, (B) proportion of individuals sampled, and (C) H3-specific antibody prevalence (± 95% CI) per month are depicted.

During the 2010 LPAIV epizootic, NP-specific LPAIV antibody prevalence increased from September onwards to 60% in December (Figure S1). During the H3 LPAIV epizootic, the proportion of local and distant migrants with H3-specific antibodies (3 of 106, 3% and 4 of 96, 4% respectively) was similar to that in residents (2 of 118, 2%; X^2^ = 0.543, p = 0.762; Figure 3). There were no differences in H3-specific antibodies between months (X^2^ = 6.996, p = 0.136). During the H3 LPAIV epizootic, H3-specific antibodies were detected on four sampling dates. On the 5^th^ of August, before the start of the H3 LPAIV epizootic, one distant migrant had H3-specific antibodies (while distant migrants constituted 14% of the sampled mallard population). During the H3 LPAIV epizootic, the first resident with H3-specific antibodies was sampled on the 21^st^ of September, with 9% of the sampled mallard population comprised of residents. After the peak of the H3 LPAIV epizootic (1^st^ of November), two local migrants, one distant migrant and one resident had antibodies specific for H3 LPAIV. That day, local migrants constituted the largest proportion of the sampled mallard population (71%). At the end of the epizootic (21^st^ of December), only migrants (local migrant: 1, distant migrant: 2) had specific antibodies against H3 LPAIV (constituting 38% and 44% of the sampled mallard population, respectively).

### Virus kinship

**Figure 4:**
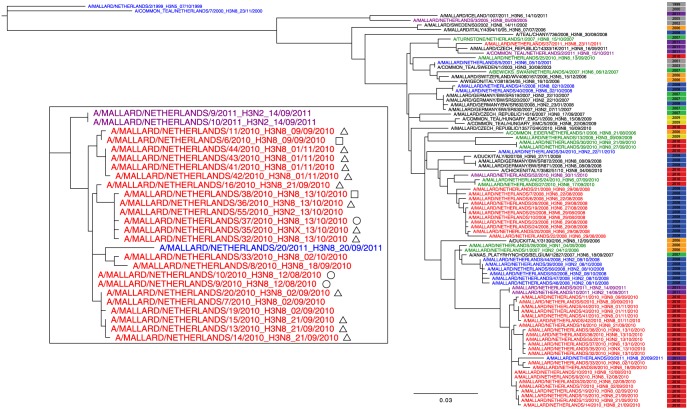
Phylogenetic analysis of HA gene of H3 low pathogenic avian influenza virus (LPAIV) isolated during the H3 LPAIV epizootic in 2010. The Maximum Likelihood (ML) tree contains samples of wild birds collected at various locations in and outside the Netherlands from 1999 until 2011. Each sampling location within the Netherlands is grouped by colour: Oud Alblas (red); Berkenwoude (blue); Lekkerkerk aan de IJssel, Oudeland van Strijen and Vlist (purple); Schiermonnikoog, Vlieland, Westerland and Wieringen (green). Locations are closely situated to the study site (i.e. duck decoy near Oud Alblas), except the locations shown in green, which are located at the coast. Year of virus isolation is listed next to isolate and grouped by colour. Detail of ML tree contains samples of the H3 LPAIV epizootic described in this study and migratory strategy of mallards: residents (RES; circle), local migrants (LM; triangle) and distant migrants (DM; square).

The HA gene sequences of the H3 LPAIV strains isolated from free-living mallards during the H3 LPAIV epizootic were monophyletic, suggesting the outbreak resulted from a single virus introduction. Although migratory mallards kept arriving at the study site during the H3 LPAIV epizootic, the genetic analysis indicates that no other H3 LPAIVs were introduced. The estimated time to the most recent common ancestor of the H3 LPAIV strains of the epizootic was spring 2009 (TMRCA 12 May 2009, LHPD95% 1 July 2008, UHPD95% 18 November 2009). The H3 LPAIV strain detected in a single mallard at our study site prior to the H3 LPAIV epizootic (10^th^ of February 2010) differed from the H3 LPAIV strains of the epizootic (HA could only be sequenced partially and is not shown in the tree), and was therefore unlikely to have seeded the outbreak. Furthermore, the H3 LPAIV strains isolated during the H3 LPAIV epizootic were not closely related to isolates obtained from mallards at our study site in autumn 2008 (sequence identity 0.958–0.967), or November 2011 (sequence identity 0.954–0.957; Figure 4). However, the H3 LPAIV strains isolated from the H3 LPAIV epizootic were genetically closely related to H3 isolates from mallards at two sampling sites 8 to 12 km away from the study site one year later, in autumn 2011 (i.e. locations Berkenwoude and Vlist; Figure 4).

H3 LPAIV strains isolated from the resident, local and distant migratory population belonged to the same cluster with little variation in nucleotide sequences (sequence identity 0.995–1; detail of Figure 4). No consistent substitutions were detected in the nucleotide sequences that correlated with the migratory strategy of birds. Evolutionary divergence of the HA of H3 LPAIV was 2.5^e-3^ nucleotide substitutions per site per year, which is lower than reported by Hill et al. [18]: 1.38 (±0.40)^e-2^.

## Discussion

Studying the role of resident and migratory hosts in the spread and circulation of pathogens in animal populations is crucial for increasing our understanding of the ecology and epidemiology of infectious diseases in wildlife. We studied virus and antibody prevalence in free-living mallards during an autumn LPAIV epizootic of subtype H3 at a local scale, focussing on the distinct role that resident and migratory hosts might have played in the introduction and circulation of this virus subtype. Although alternative interpretations cannot be entirely excluded, our findings suggest that the H3 LPAIV causing the epizootic was present in resident mallards prior to the arrival of migrants, followed by virus amplification importantly associated with the arrival of migratory mallards.

H3 LPAIV isolations from residents, local and distant migrants belonged to the same genetic cluster (Figure 4). However, we cannot fully exclude the possibility that novel introductions of H3 LPAIV, or other LPAIV HA subtypes, by migratory birds occurred that were subsequently outcompeted by the dominant epizootic H3 LPAIV strain and thus remained undetected during our monitoring (i.e. competitive exclusion principle; [42]). For instance, another H3 LPAIV epizootic in the area (i.e. Berkenwoude in 2008) resulted from multiple virus introductions. The H3 LPAIV that induced the 2010 epizootic was closely related to H3 LPAIV strains isolated in the near surrounding one year after the epizootic (i.e. Berkenwoude and Vlist in 2011). This suggests that after the epizootic H3 LPAIV may have overwintered and had been maintained locally. H3 virus prevalence in migratory mallards was higher (especially in distant migrants) and more prolonged (especially in local migrants) than in resident individuals. This finding corresponds with the results of van Dijk et al. [19] who found a three-fold increase in overall (i.e. non LPAIV-subtype specific) virus prevalence in migratory mallards. However, during the peak of the H3 LPAIV epizootic many residents were also infected with H3 LPAIV, which may be a consequence of the local amplification and increased viral deposition in the environment (i.e. water and sediment) at the study site. The local amplification may thus be a self-reinforcing process.

At the start of the H3 LPAIV epizootic, almost exclusively resident birds were infected with H3 LPAIV. However, it is not surprising that the majority of H3 LPAIV infections were found in residents, since the sampled mallard population consisted mainly out of resident birds (88%). What is remarkable though is that one week after detection of the first H3 LPAIV infections, no migrants were infected while a large proportion of the sampled mallard population consisted of migrants (∼40%). Either migratory birds were not, or to a lesser extent, susceptible to H3 LPAIV infection, or contact rates and the amount of H3 virus particles in the surface water were still too low to infect arriving migrants. Interestingly, the peak of virus infection in October in the resident population was mainly induced by recaptured resident birds (i.e. captured multiple times) (Figure S2). H3 virus prevalence in primary residents (i.e. captured for the first time) remained relatively low and increased in December. Potentially recaptured residents were trap-prone and had a higher probability of being exposed—and consequently becoming infected—than primary residents. In addition, in October the population of recaptured residents sampled was three-times higher than the population of primary residents sampled, increasing the probability of virus detection in recaptured residents.

During the H3 LPAIV epizootic, H3-specific antibodies were detected in both resident and migratory mallards, albeit in very few individuals and at low titres. A week before the start of the H3 LPAIV epizootic, H3-specific antibodies were found in a distant migrant (5^th^ of August). We cannot exclude that this individual was infected with H3 LPAIV either during migration, at a stop-over site or at the breeding grounds. Hypothetically, this individual could have been infected with H3 LPAIV when transiting through southern Sweden (i.e. feather hydrogen stable isotope -129.2‰ suggest it originated from southern Scandinavia, Baltic States or Russia; [33]), introducing this virus to the wintering grounds. H3 LPAIV is detected frequently in mallards sampled in southern Sweden in early autumn [43]. Although our genetic analysis does not support this theory, it should be noted that only few H3 LPAIV originating from Sweden or other northern European countries were available and were included in the genetic analysis.

Several local and distant migrants had H3-specific antibodies after the peak of the H3 LPAIV epizootic. Since these birds were captured once during the H3 LPAIV epizootic, we cannot exclude that an H3 LPAIV infection outside the study site triggered this antibody response (i.e. genetically different H3 LPAIV were isolated at other locations in the Netherlands). Resident mallards with H3-specific antibodies most likely have been infected by the H3 LPAIV of the epizootic. Only 20% (1 of 5) of residents that had been infected with H3 LPAIV during the epizootic had H3-specific antibodies when recaptured (i.e. recaptured within 31 days since longevity of detectable HA specific antibodies is short; [44]). As result of H3 LPAIV infection an H3 specific antibody titre may have been generated, yet not detected due to antibody dynamics and timing of sampling, and/or sensitivity of the HI assay.

In conclusion, by combining virology, serology and phylogeny analyses with stable isotopes we demonstrate that a local H3 LPAIV epizootic in mallards was likely induced by a single virus introduction into susceptible residents, followed by a period of local virus amplification that was associated with the influx of migratory mallards. In addition to the study of Hill et al. [18], who showed long-distance movement of LPAIV genes by migrating mallards on a macro-ecological scale, we showed an association between local amplification of H3 LPAIV and the arrival of migratory mallards at the wintering grounds at a much smaller ecological scale. We suggest an additional role for migrating mallards as local amplifiers, based on the difference in H3 LPAIV prevalence between resident and migratory mallards upon arrival at the wintering grounds. This study exemplifies the difficulty of elucidating the role of migratory and resident hosts in infectious disease dynamics in wildlife, but provides encouraging indications that the here presented multifaceted approach may open a window on these processes.

## Supporting Information

Figure S1 **Prevalence of avian influenza-specific antibodies in free-living mallards during H3 epizootic.** This figure shows prevalence of avian influenza virus nucleoprotein (NP)-specific antibodies in mallards (*Anas platyrhynchos*) during the H3 low pathogenic avian influenza virus epizootic in 2010.
(PDF)

Figure S2 **Prevalence of H3 influenza virus in resident and migratory mallards during H3 epizootic.** This figure shows H3 low pathogenic avian influenza virus (LPAIV) prevalence in resident mallards (i.e. primary captured and recaptured), local and distant migratory mallards, during the H3 LPAIV epizootic in 2010.
(PDF)

Table S1 **The H3 influenza virus strain names and accession numbers used in this study.** This table includes all H3 influenza virus strain names and accession numbers used in this study.
(PDF)

Table S2 **The sequence information of H3 influenza viruses from GISAID's EpiFlu Database.** This table includes details of H3 influenza viruses downloaded from GISAID's EpiFlu Database.
(PDF)

Table S3 **Sample collection for influenza virus and antibody detection from free-living mallards.** This table includes number of samples collected for influenza virus and antibody detection from free-living mallards (*Anas platyrhynchos*) during the H3 low pathogenic avian influenza virus epizootic.
(PDF)

## References

[1] Altizer S, Bartel R, Han BA (2011) Animal migration and infectious disease risk. Science 331: 296–302 10.1126/science.1194694 21252339

[2] Rappole JH, Hubálek Z (2003) Migratory birds and West Nile virus. J Appl Microbiol 94: 47S–58S 10.4269/ajtmh.2009.09-0106 12675936

[3] Leroy EM, Epelboin A, Mondonge V, Pourrut X, Gonzalez JP, et al (2009) Human Ebola outbreak resulting from direct exposure to fruit bats in Luebo, Democratic Republic of Congo, 2007. Vector-Borne Zoonotic Dis 9: 723–728 10.1089/vbz.2008.0167 19323614

[4] Leighton FA (2002) Health risk assessment of the translocation of wild animals. Rev Sci Tech Off Int Epizoot 21: 187–195. 11974629

[5] Waldenström J, Bensch S, Kiboi S, Hasselquist D, Ottosson U (2002) Cross-species infection of blood parasites between resident and migratory songbirds in Africa. Mol Ecol 11: 1545–1554 10.1046/j.1365-294X.2002.01523.x 12144673

[6] Haydon DT, Cleaveland S, Taylor LH, Laurenson MK (2002) Identifying reservoirs of infection: aconceptual and practical challenge. Emerg Infect Dis 8: 1468–1473. 12498665 10.3201/eid0812.010317 PMC2738515

[7] Webster RG, Bean WJ, Gorman OT, Chambers TM, Kawaoka Y (1992) Evolution and ecology of influenza A viruses. Microbiol Rev 56: 152–179. 1579108 10.1128/mr.56.1.152-179.1992 PMC372859

[8] Daoust PY, van de Bildt M, van Riel D, van Amerongen G, Bestebroer T, et al (2012) Replication of 2 subtypes of low-pathogenicity avian influenza virus of duck and gull origins in experimentally infected mallard ducks. Vet Pathol 50: 548–559 10.1177/0300985812469633 23242805

[9] Höfle U, van de Bildt MWG, Leijten LM, van Amerongen G, Verhagen JH, et al (2012) Tissue tropism and pathology of natural influenza virus infection in black-headed gulls (*Chroicocephalus ridibundus*). Avian Pathol 41: 547–553 10.1080/03079457.2012.744447 23237367

[10] Kuiken T (2013) Is low pathogenic avian influenza virus virulent for wild waterbirds? Proc R Soc B-Biol Sci 280: 20130990 10.1098/rspb.2013.0990 PMC3774239 23740783

[11] Olsen B, Munster VJ, Wallensten A, Waldenström J, Osterhaus ADME, et al (2006) Global patterns of influenza A virus in wild birds. Science 312: 384–388 10.1126/science.1122438 16627734

[12] Tong SX, Li Y, Rivailler P, Conrardy C, Castillo DAA, et al (2012) A distinct lineage of influenza A virus from bats. Proc Natl Acad Sci U S A 109: 4269–4274 10.1073/pnas.1116200109 22371588 PMC3306675

[13] Tong S, Zhu X, Li Y, Shi M, Zhang J, et al (2013) New world bats harbor diverse influenza A viruses. Plos Pathog 9: e1003657 10.1371/journal.ppat.1003657 24130481 PMC3794996

[14] Stallknecht DE, Shane SM, Zwank PJ, Senne DA, Kearney MT (1990) Avian influenza viruses from migratory and resident ducks of coastal Louisiana. Avian Dis 34: 398–405. 2369380

[15] Ferro PJ, Budke CM, Peterson MJ, Cox D, Roltsch E, et al (2010) Multiyear surveillance for avian influenza virus in waterfowl from wintering grounds, Texas coast, USA. Emerg Infect Dis 16: 1224–1230 10.3201/eid1608.091864 20678315 PMC3298295

[16] Lack D (1943) The problem of partial migration. Br Birds 37: 122–130.

[17] Chapman BB, Brönmark C, Nilsson JA, Hansson LA (2011) The ecology and evolution of partial migration. Oikos 120: 1764–1775 10.1111/j.1600-0706.2011.20131.x

[18] Hill NJ, Takekawa JY, Ackerman JT, Hobson KA, Herring G, et al (2012) Migration strategy affects avian influenza dynamics in mallards (*Anas platyrhynchos*). Mol Ecol 21: 5986–5999 10.1111/j.1365-294X.2012.05735.x 22971007

[19] van Dijk JGB, Hoye BJ, Verhagen JH, Nolet BA, Fouchier RAM, et al (2014) Juveniles and migrants as drivers for seasonal epizootics of avian influenza virus. J Anim Ecol 83: 266–275 10.1111/1365-2656.12131 24033258 PMC3869896

[20] Latorre-Margalef N, Gunnarsson G, Munster VJ, Fouchier RAM, Osterhaus ADME, et al (2009) Effects of influenza A virus infection on migrating mallard ducks. Proc R Soc B-Biol Sci 276: 1029–1036 10.1098/rspb.2008.1501 PMC2679067 19129127

[21] Hénaux V, Samuel MD (2011) Avian influenza shedding patterns in waterfowl: implications for surveillance, environmental transmission, and disease spread. J Wildl Dis 47: 566–578. 21719821 10.7589/0090-3558-47.3.566

[22] Krauss S, Walker D, Pryor SP, Niles L, Li CH, et al (2004) Influenza A viruses of migrating wild aquatic birds in North America. Vector-Borne Zoonotic Dis 4: 177–189 10.1089/1530366042162452 15631061

[23] Munster VJ, Baas C, Lexmond P, Waldenström J, Wallensten A, et al (2007) Spatial, temporal, and species variation in prevalence of influenza A viruses in wild migratory birds. PLoS Pathog 3: 630–638 10.1371/journal.ppat.0030061 PMC1876497 17500589

[24] Scott DA, Rose PM (1996) Atlas of Anatidae Populations in Africa and Western Eurasia, Wetlands International Publication No. 41. Wageningen: Wetlands International. 336 p.

[25] Payne-Gallwey R (1886) The book of duck decoys, their construction, management, and history. London: J. van Voorst. 154 p.

[26] Boyd H, Harrison J, Allison A (1975) Duck wings: a study of duck production. A WAGBI Publication. Chester: Marley Ltd., and the Harrison Zoological Museum. 112 p.

[27] Fouchier RAM, Munster VJ (2009) Epidemiology of low pathogenic avian influenza viruses in wild birds. Rev Sci Tech Off Int Epizoot 28: 49–58. 10.20506/rst.28.1.1863 19618618

[28] Munster VJ, Baas C, Lexmond P, Bestebroer TM, Guldemeester J, et al (2009) Practical considerations for high-throughput Influenza A virus surveillance studies of wild birds by use of molecular diagnostic tests. J Clin Microbiol 47: 666–673 10.1128/jcm.01625-08 19109483 PMC2650931

[29] Hoye BJ (2012) Variation in postsampling treatment of avian blood affects ecophysiological interpretations. Methods Ecol Evol 3: 162–167 10.1111/j.2041-210X.2011.00135.x

[30] Peterson BJ, Fry B (1987) Stable isotopes in ecosystem studies. Annu Rev Ecol Syst 18: 293–320 10.1146/annurev.ecolsys.18.1.293

[31] Hobson KA (1999) Tracing origins and migration of wildlife using stable isotopes: a review. Oecologia 120: 314–326 10.1007/s004420050865 28308009

[32] Hobson KA, Wassenaar LI (1997) Linking breeding and wintering grounds of neotropical migrant songbirds using stable hydrogen isotopic analysis of feathers. Oecologia 109: 142–148 10.1007/s004420050068 28307604

[33] van Dijk JGB, Meissner W, Klaassen M (2014) Improving provenance studies in migratory birds when using feather hydrogen stable isotopes. J Avian Biol 45: 103–108 10.1111/j.1600-048X.2013.00232.x

[34] Fouchier RAM, Schneeberger PM, Rozendaal FW, Broekman JM, Kemink SAG, et al (2004) Avian influenza A virus (H7N7) associated with human conjunctivitis and a fatal case of acute respiratory distress syndrome. Proc Natl Acad Sci U S A 101: 1356–1361 10.1073/pnas.0308352100 14745020 PMC337057

[35] Hoffmann E, Stech J, Guan Y, Webster RG, Perez DR (2001) Universal primer set for the full-length amplification of all influenza A viruses. Arch Virol 146: 2275–2289 10.1007/s007050170002 11811679

[36] Posada D (2008) jModelTest: Phylogenetic model averaging. Mol Biol Evol 25: 1253–1256 10.1093/molbev/msn083 18397919

[37] Westgeest KB, Russell CA, Lin XD, Spronken MIJ, Bestebroer TM, et al (2014) Genomewide analysis of reassortment and evolution of human influenza A(H3N2) viruses circulating between 1968 and 2011. J Virol 88: 2844–2857 10.1128/jvi.02163-13 24371052 PMC3958060

[38] Hirst GK (1943) Studies of antigenic differences among strains of influenza a by means of red cell agglutination. J Exp Med 78: 407–423 10.1084/jem.78.5.407 19871338 PMC2135416

[39] R Development Core Team (2012) R: A Language and Environment for Statistical Computing. Vienna, Austria: R Foundation for Statistical Computing.

[40] Bates D, Maechler M, Bolker B (2012) Lme4:linear mixed-effects models using S4 classes. R package version 0.999999-0. http://CRAN.R-project.org/package=lme4.

[41] Hothorn T, Bretz F, Westfall P (2008) Simultaneous inference in general parametric models. Biometrical J 50: 346–363 10.1002/bimj.200810425 18481363

[42] Hardin G (1960) Competitive exclusion principle. Science 131: 1292–1297 10.1126/science.131.3409.1292 14399717

[43] Latorre-Margalef N, Tolf C, Grosbois V, Avril A, Bengtsson D, et al (2014) Long-term variation in influenza A virus prevalence and subtype diversity in migratory mallards in northern Europe. Proc R Soc B-Biol Sci 281: 20140098 10.1098/rspb.2014.0098 PMC3953854 24573857

[44] Curran JM, Robertson ID, Ellis TM, Selleck PW, O'Dea MA (2013) Variation in the responses of wild species of duck, gull, and wader to inoculation with a wild-bird-origin H6N2 low pathogenicity avian influenza virus. Avian Dis 57: 581–586. 24283122 10.1637/10458-112712-Reg.1

