# Supplementary material for: Migratory Birds Reinforce Local Circulation of Avian Influenza Viruses
Source: PLoS One. 2014 Nov 12;9(11):e112366. doi: 10.1371/journal.pone.0112366 (PMC4229208; doi:10.1371/journal.pone.0112366)
Supplement: Figure S1 — Prevalence of avian influenza-specific antibodies in free-living mallards during H3 epizootic. This figure shows prevalence of avian influenza virus nucleoprotein (NP)-specific antibodies in mallards (Anas platyrhynchos) during the H3 low pathogenic avian influenza virus epizootic in 2010. (PDF) [file pone.0112366.s001.pdf]

Supporting Information

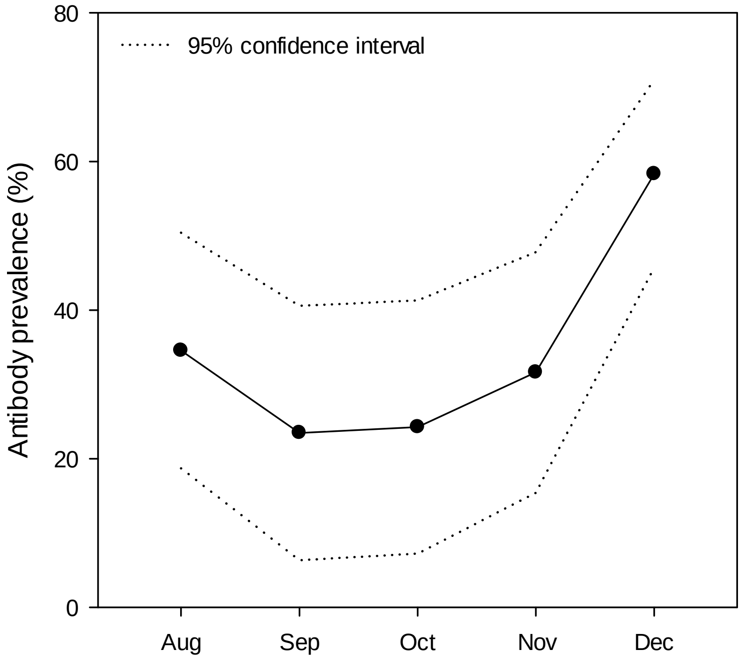

Figure S1. Monthly prevalence (± 95% CI) of avian influenza virus (AIV) NP specific antibodies in mallards (*Anas platyrhynchos*) during the H3 low pathogenic avian influenza virus (LPAIV) epizootic in 2010.
