# Supplementary material for: Migratory Birds Reinforce Local Circulation of Avian Influenza Viruses
Source: PLoS One. 2014 Nov 12;9(11):e112366. doi: 10.1371/journal.pone.0112366 (PMC4229208; doi:10.1371/journal.pone.0112366)
Supplement: Figure S2 — Prevalence of H3 influenza virus in resident and migratory mallards during H3 epizootic. This figure shows H3 low pathogenic avian influenza virus (LPAIV) prevalence in resident mallards (i.e. primary captured and recaptured), local and distant migratory mallards, during the H3 LPAIV epizootic in 2010. (PDF) [file pone.0112366.s002.pdf]

Supporting Information

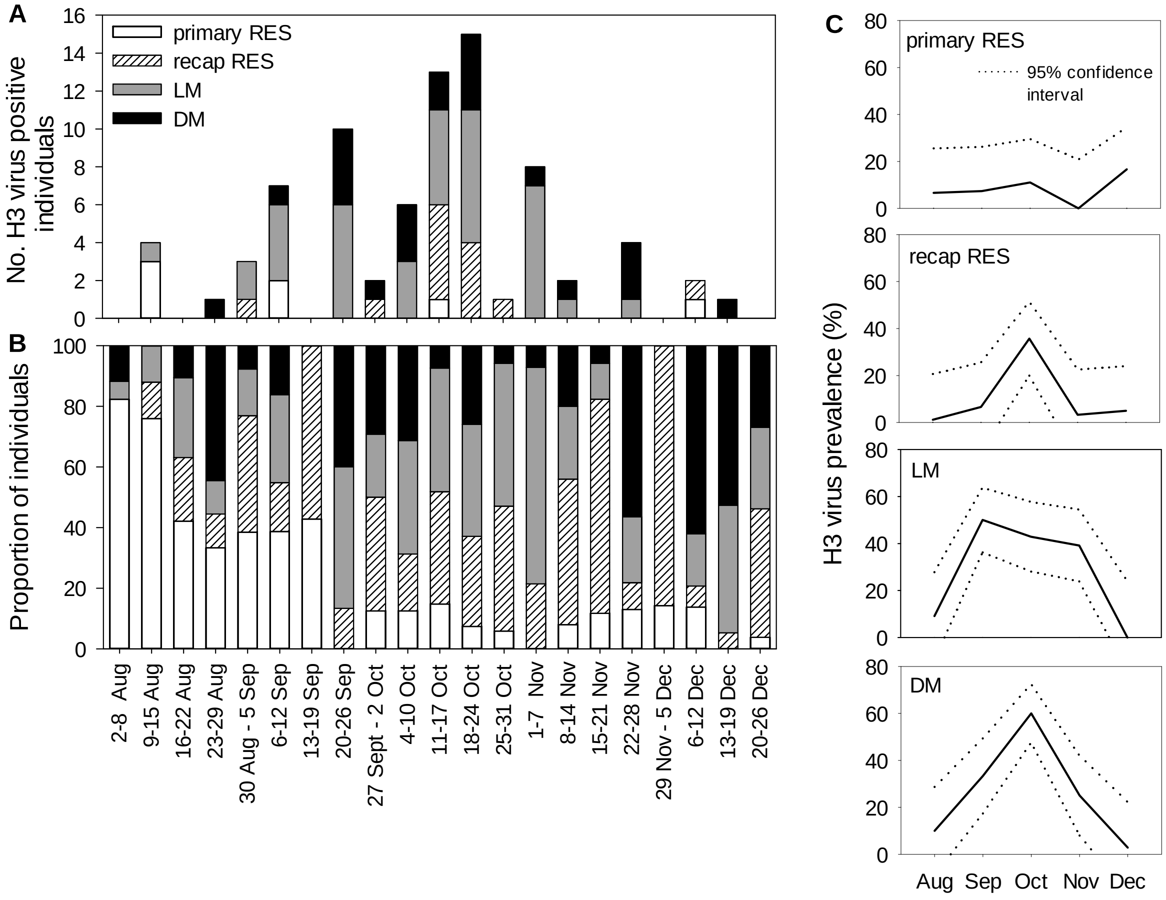

Figure S2. H3 low pathogenic avian influenza virus (LPAIV) prevalence in residents, local and distant migratory mallards during the H3 epizootic in 2010. For residents that were first captured (primary RES), recaptured residents (recap RES), local migrants (LM) and distant migrants (DM) the (A) number of H3 virus positive individuals per week, (B) proportion of individuals sampled per week, and (C) H3 virus prevalence (± 95% CI) per month are depicted.

.
