# Supplementary material for: Migratory Birds Reinforce Local Circulation of Avian Influenza Viruses
Source: PLoS One. 2014 Nov 12;9(11):e112366. doi: 10.1371/journal.pone.0112366 (PMC4229208; doi:10.1371/journal.pone.0112366)
Supplement: Table S1 — The H3 influenza virus strain names and accession numbers used in this study. This table includes all H3 influenza virus strain names and accession numbers used in this study. (PDF) [file pone.0112366.s003.pdf]

Supporting Information

Table S1. List of virus strain names and accession numbers of H3 influenza A viruses included in this study as listed in online databases Influenza Research Database (IRD) (http://www.fludb.org) and GISAID EpiFlu (http://platform.gisiad.org).

Virus strain name	Accession no.	Virus strain name	Accession no.
A/Bewick's Swan/Netherlands/4/2007(H3N6)	IRDAccession_1099728	A/Mallard/Netherlands/20/2010(H3N8)	IRDAccession_1099798
A/Common Teal/Hungary-EMC/1/2009(H3N8)	IRDAccession_1099753	A/Mallard/Netherlands/11/2010(H3N8)	IRDAccession_1099799
A/Common Teal/Hungary-EMC/5/2009(H3N8)	IRDAccession_1099754	A/Mallard/Netherlands/6/2010(H3N8)	IRDAccession_1099800
A/Mallard/Netherlands/52/2010(H3N8)	IRDAccession_1099755	A/Mallard/Netherlands/15/2010(H3N8)	IRDAccession_1099801
A/Common Teal/Netherlands/2/2011(H3N8)	IRDAccession_1099756	A/Mallard/Netherlands/35/2010(H3Nx)	IRDAccession_1099802
A/Mallard/Netherlands/6/2008(H3N6)	IRDAccession_1099759	A/Mallard/Netherlands/32/2010(H3N8)	IRDAccession_1099803
A/Mallard/Netherlands/7/2008(H3N8)	IRDAccession_1099760	A/Mallard/Netherlands/38/2010(H3N8)	IRDAccession_1099804
A/Mallard/Netherlands/10/2008(H3N6)	IRDAccession_1099761	A/Mallard/Netherlands/36/2010(H3N8)	IRDAccession_1099805
A/Mallard/Netherlands/56/2008(H3N2)	IRDAccession_1099762	A/Mallard/Netherlands/41/2010(H3N8)	IRDAccession_1099806
A/Mallard/Netherlands/39/2008(H3N2)	IRDAccession_1099763	A/Mallard/Netherlands/42/2010(H3N8)	IRDAccession_1099807
A/Mallard/Netherlands/40/2008(H3N6)	IRDAccession_1099764	A/Mallard/Netherlands/43/2010(H3N8)	IRDAccession_1099808
A/Mallard/Netherlands/41/2008(H3N8)	IRDAccession_1099765	A/Mallard/Netherlands/44/2010(H3N8)	IRDAccession_1099809
A/Mallard/Netherlands/44/2008(H3N2)	IRDAccession_1099767	A/Mallard/Netherlands/10/2010(H3N8)	IRDAccession_1099810
A/Mallard/Netherlands/19/2010(H3N8)	IRDAccession_1099768	A/Mallard/Netherlands/37/2010(H3N8)	IRDAccession_1099811
A/Mallard/Netherlands/7/2010(H3N8)	IRDAccession_1099769	A/Mallard/Netherlands/55/2010(H3N2)	IRDAccession_1099812
A/Mallard/Netherlands/13/2010(H3N8)	IRDAccession_1099770	A/Anas_platyrhynchos/Belgium/12827/2007(H3N8)	EPI_ISL_26267
A/Mallard/Netherlands/14/2010(H3N8)	IRDAccession_1099771	A/mallard/Germany-BW/SR872/2008(H3N8)	EPI_ISL_79643
A/Mallard/Netherlands/16/2010(H3N8)	IRDAccession_1099772	A/mallard/Germany-BW/SR871/2008(H3N8)	EPI_ISL_79642
A/Mallard/Netherlands/13/2009(H3N3)	IRDAccession_1099773	A/mallard/Germany-BW/SR632/2008(H3N2)	EPI_ISL_79640
A/Mallard/Netherlands/9/2010(H3N8)	IRDAccession_1099774	A/mallard/Germany-BW/SR530/2007(H3N2)	EPI_ISL_79639
A/Mallard/Netherlands/27/2010(H3N8)	IRDAccession_1099775	A/mallard/Germany-BW/SR520/2007(H3N2)	EPI_ISL_79638
A/Mallard/Netherlands/30/2010(H3N8)	IRDAccession_1099776	A/mallard/Germany-BW/SR519/2007(H3N2)	EPI_ISL_79637
A/Mallard/Netherlands/39/2010(H3N8)	IRDAccession_1099777	A/common_teal/Netherlands/7/2000(H3N8)	EPI_ISL_15008
A/Mallard/Netherlands/24/2010(H3N6)	IRDAccession_1099778	A/mallard/Iceland/1007/2011(H3N6)	EPI_ISL_148200
A/Mallard/Netherlands/25/2010(H3N8)	IRDAccession_1099779	A/mallard/Czech_Republic/14333-1K/2011(H3N8)	EPI_ISL_116136
A/Mallard/Netherlands/34/2010(H3N2)	IRDAccession_1099780	A/mallard/Czech_Republic/14516/2007(H3N8)	EPI_ISL_63529
A/Mallard/Netherlands/8/2010(H3N8)	IRDAccession_1099781	A/mallard/Sweden/50/2002(H3N8)	EPI_ISL_73381
A/Mallard/Netherlands/33/2010(H3N8)	IRDAccession_1099782	A/mallard/Netherlands/5/2001(H3N6)	EPI_ISL_73371
A/Mallard/Netherlands/9/2011(H3N2)	IRDAccession_1099783	A/mallard/Netherlands/2/1999(H3N5)	EPI_ISL_73370
A/Mallard/Netherlands/10/2011(H3N2)	IRDAccession_1099784	A/common_teal/Sweden/1/2003(H3N3)	EPI_ISL_73363
A/Mallard/Netherlands/20/2011(H3N8)	IRDAccession_1099785	A/mallard/Netherlands/1/2007(H3N2)	EPI_ISL_33850
A/Mallard/Netherlands/37/2011(H3N8)	IRDAccession_1099786	A/mallard/Switzerland/WV4060167/2006(H3N5)	EPI_ISL_33832
A/Mallard/Netherlands/19/2008(H3N6)	IRDAccession_1099787	A/turnstone/Netherlands/1/2007(H3N8)	EPI_ISL_30805
A/Mallard/Netherlands/20/2008(H3N6)	IRDAccession_1099788	A/common_eider/Netherlands/1/2006(H3N8)	EPI_ISL_30804
A/Mallard/Netherlands/21/2008(H3N8)	IRDAccession_1099789	A/mallard/Netherlands/3/2005(H3N8)	EPI_ISL_30793
A/Mallard/Netherlands/22/2008(H3N6)	IRDAccession_1099790	A/teal/Chany/736/2008(H3N8)	EPI_ISL_97501
A/Mallard/Netherlands/23/2008(H3N6)	IRDAccession_1099791	A/mallard/Czech_Republic/13577-24K/2010(H3N8)	EPI_ISL_89980
A/Mallard/Netherlands/24/2008(H3N8)	IRDAccession_1099792	A/mallard/Netherlands/28/2006(H3N1)	EPI_ISL_84553
A/Mallard/Netherlands/25/2008(H3N6)	IRDAccession_1099793	A/wigeon/Italy/3818-34/05(H3N8)	EPI_ISL_85911
A/Mallard/Netherlands/26/2008(H3N6)	IRDAccession_1099794	A/mallard/Italy/4394-10/05(H3N8)	EPI_ISL_85910
A/Mallard/Netherlands/47/2008(H3N2)	IRDAccession_1099795	A/chicken/Italy/3582-51/10(H3N8)	EPI_ISL_85902
A/Mallard/Netherlands/48/2008(H3N2)	IRDAccession_1099796	A/duck/Italy/3139-2/06(H3N8)	EPI_ISL_85901
A/Mallard/Netherlands/50/2008(H3N2)	IRDAccession_1099797	A/duck/Italy/6207/08(H3N6)	EPI_ISL_85900
