# Supplementary material for: Migratory Birds Reinforce Local Circulation of Avian Influenza Viruses
Source: PLoS One. 2014 Nov 12;9(11):e112366. doi: 10.1371/journal.pone.0112366 (PMC4229208; doi:10.1371/journal.pone.0112366)
Supplement: Table S2 — The sequence information of H3 influenza viruses from GISAID's EpiFlu Database. This table includes details of H3 influenza viruses downloaded from GISAID's EpiFlu Database. (PDF) [file pone.0112366.s004.pdf]

Supporting Information

Table S2. Low pathogenic avian influenza virus sequence information. We gratefully acknowledge the authors, originating and submitting laboratories of the sequences from GISAID's EpiFlu™ Database on which this research is based. All submitters of data may be contacted directly via the GISAID website. [1]Veterinary and Agrochemical Research Institute, Uccle, Belgium, [2]Friedrich-Loeffler-Institut, Riems, Germany, [3]Istituto Zooprofilattico Sperimentale Delle Venezie, Venice, Italy.

Segment ID	Segment	Country	Collection date	Isolate name	Originating laboratory	Submitting laboratory	Authors
EPI_ISL_26267	HA	Belgium	9/18/07	A/Anas_platyrhynchos/Belgium/12827/2007(H3N8)	CODA-CERVA[1]	CODA-CERVA[1]	Thierry van den Berg
EPI_ISL_79643	HA	Germany	9/8/08	A/mallard/Germany-BW/SR872/2008(H3N8)	FLI[2]	FLI[2]	Elke Starick
EPI_ISL_79642	HA	Germany	9/8/08	A/mallard/Germany-BW/SR871/2008(H3N8)	FLI[2]	FLI[2]	Elke Starick
EPI_ISL_79640	HA	Germany	1/23/08	A/mallard/Germany-BW/SR632/2008(H3N2)	FLI[2]	FLI[2]	Elke Starick
EPI_ISL_79639	HA	Germany	11/7/07	A/mallard/Germany-BW/SR530/2007(H3N2)	FLI[2]	FLI[2]	Elke Starick
EPI_ISL_79638	HA	Germany	10/22/07	A/mallard/Germany-BW/SR520/2007(H3N2)	FLI[2]	FLI[2]	Elke Starick
EPI_ISL_79637	HA	Germany	10/22/07	A/mallard/Germany-BW/SR519/2007(H3N2)	FLI[2]	FLI[2]	Elke Starick
EPI_ISL_15008	HA	Netherlands	11/23/00	A/common_teal/Netherlands/7/2000(H3N8)			
EPI_ISL_148200	HA	Iceland	10/14/11	A/mallard/Iceland/1007/2011(H3N6)			
EPI_ISL_116136	HA	Czech Republic	9/16/11	A/mallard/Czech_Republic/14333-1K/2011(H3N8)			
EPI_ISL_63529	HA	Czech Republic	9/17/11	A/mallard/Czech_Republic/14516/2007(H3N8)			
EPI_ISL_73381	HA	Sweden	11/14/02	A/mallard/Sweden/50/2002(H3N8)			
EPI_ISL_73371	HA	Netherlands	10/5/01	A/mallard/Netherlands/5/2001(H3N6)			
EPI_ISL_73370	HA	Netherlands	10/7/99	A/mallard/Netherlands/2/1999(H3N5)			
EPI_ISL_73363	HA	Sweden	8/30/03	A/common_teal/Sweden/1/2003(H3N3)			
EPI_ISL_33850	HA	Netherlands	12/4/06	A/mallard/Netherlands/1/2007(H3N2)			
EPI_ISL_33832	HA	Switzerland	12/15/06	A/mallard/Switzerland/WV4060167/2006(H3N5)			
EPI_ISL_30805	HA	Netherlands	10/15/07	A/turnstone/Netherlands/1/2007(H3N8)			
EPI_ISL_30804	HA	Netherlands	8/5/06	A/common_eider/Netherlands/1/2006(H3N8)			
EPI_ISL_30793	HA	Netherlands	9/5/05	A/mallard/Netherlands/3/2005(H3N8)			
EPI_ISL_97501	HA	Russia	8/30/08	A/teal/Chany/736/2008(H3N8)			
EPI_ISL_89980	HA	Czech Republic	9/16/10	A/mallard/Czech_Republic/13577-24K/2010(H3N8)			
EPI_ISL_84553	HA	Netherlands	9/4/06	A/mallard/Netherlands/28/2006(H3N1)			
EPI_ISL_85911	HA	Italy	10/16/06	A/wigeon/Italy/3818-34/05(H3N8)	IZSV[3]	IZSV[3]	Isabella Monne
EPI_ISL_85910	HA	Italy	7/7/06	A/mallard/Italy/4394-10/05(H3N8)	IZSV[3]	IZSV[3]	Isabella Monne
EPI_ISL_85902	HA	Italy	6/4/10	A/chicken/Italy/3582-51/10(H3N8)	IZSV[3]	IZSV[3]	Isabella Monne
EPI_ISL_85901	HA	Italy	9/12/06	A/duck/Italy/3139-2/06(H3N8)	IZSV[3]	IZSV[3]	Isabella Monne
EPI_ISL_85900	HA	Italy	11/27/08	A/duck/Italy/6207/08(H3N6)	IZSV[3]	IZSV[3]	Isabella Monne
