# Supplementary material for: Migratory Birds Reinforce Local Circulation of Avian Influenza Viruses
Source: PLoS One. 2014 Nov 12;9(11):e112366. doi: 10.1371/journal.pone.0112366 (PMC4229208; doi:10.1371/journal.pone.0112366)
Supplement: Table S3 — Sample collection for influenza virus and antibody detection from free-living mallards. This table includes number of samples collected for influenza virus and antibody detection from free-living mallards (Anas platyrhynchos) during the H3 low pathogenic avian influenza virus epizootic. (PDF) [file pone.0112366.s005.pdf]

Supporting Information

Table S3. Samples collected for virus and antibody detection from free-living mallards (*Anas platyrhynchos*) during the H3 low pathogenic avian influenza virus (LPAIV) epizootic in 2010. Samples were collected from resident birds that were first captured (primary), recaptured residents, local and distant migratory birds, and were specified by age (juvenile: <1 year, adult: >1 year) and sex.

			Resident		Local migrant	Distant migrant
	Age	Sex	Primary	Recapture		
Virology			94	55	113	98
	Juvenile	Male	9	7	25	23
		Female	8	5	8	11
	Adult	Male	42	26	31	31
		Female	35	17	49	33
Serology			79	30	106	96
	Juvenile	Male	8	5	27	25
		Female	8	4	8	11
	Adult	Male	32	14	27	30
		Female	31	7	44	30
